# COVID-19 and Heart Failure with Preserved and Reduced Ejection Fraction Clinical Outcomes among Hospitalized Patients in the United States

**DOI:** 10.3390/v15030600

**Published:** 2023-02-22

**Authors:** Adeel Nasrullah, Karthik Gangu, Harmon R. Cannon, Umair A. Khan, Nichole B. Shumway, Aneish Bobba, Shazib Sagheer, Prabal Chourasia, Hina Shuja, Sindhu Reddy Avula, Rahul Shekhar, Abu Baker Sheikh

**Affiliations:** 1 Division of Pulmonology and Critical Care, Allegheny Health Network, Pittsburg, PA 15212, USA; 2 Department of Internal Medicine, University of Kansas Medical Center, Kansas City, KS 66103, USA; 3 Department of Internal Medicine, University of New Mexico Health Sciences Center, Albuquerque, NM 87106, USA; 4 Department of Medicine, John H Stronger Hospital, Cook County, Chicago, IL 60612, USA; 5 Department of Hospital Medicine, Mary Washington Hospital, Fredericksburg, VA 22401, USA; 6 Department of Medicine, Karachi Medical and Dental College, Karachi 74700, Pakistan; 7 Department of Interventional Cardiology, Division of Cardiology, University of Kansas, St. Francis Campus, Kansas City, KS 66606, USA

**Keywords:** COVID-19, congestive heart failure, National Inpatient Sample database, mortality, HFpEF, HFrEF, United States

## Abstract

Heart failure exacerbations impart significant morbidity and mortality, however, large- scale studies assessing outcomes in the setting of concurrent coronavirus disease-19 (COVID-19) are limited. We utilized National Inpatient Sample (NIS) database to compare clinical outcomes in patients admitted with acute congestive heart failure exacerbation (CHF) with and without COVID-19 infection. A total of 2,101,980 patients (Acute CHF without COVID-19 (*n* = 2,026,765 (96.4%) and acute CHF with COVID-19 (*n* = 75,215, 3.6%)) were identified. Multivariate logistic regression analysis was utilized to compared outcomes and were adjusted for age, sex, race, income level, insurance status, discharge quarter, Elixhauser co-morbidities, hospital location, teaching status and bed size. Patients with acute CHF and COVID-19 had higher in-hospital mortality compared to patients with acute CHF alone (25.78% vs. 5.47%, adjust OR (aOR) 6.3 (95% CI 6.05–6.62, *p* < 0.001)) and higher rates of vasopressor use (4.87% vs. 2.54%, aOR 2.06 (95% CI 1.86–2.27, *p* < 0.001), mechanical ventilation (31.26% vs. 17.14%, aOR 2.3 (95% CI 2.25–2.44, *p* < 0.001)), sudden cardiac arrest (5.73% vs. 2.88%, aOR 1.95 (95% CI 1.79–2.12, *p* < 0.001)), and acute kidney injury requiring hemodialysis (5.56% vs. 2.94%, aOR 1.92 (95% CI 1.77–2.09, *p* < 0.001)). Moreover, patients with heart failure with reduced ejection fraction had higher rates of in-hospital mortality (26.87% vs. 24.5%, adjusted OR 1.26 (95% CI 1.16–1.36, *p* < 0.001)) with increased incidence of vasopressor use, sudden cardiac arrest, and cardiogenic shock as compared to patients with heart failure with preserved ejection fraction. Furthermore, elderly patients and patients with African-American and Hispanic descents had higher in-hospital mortality. Acute CHF with COVID-19 is associated with higher in-hospital mortality, vasopressor use, mechanical ventilation, and end organ dysfunction such as kidney failure and cardiac arrest.

## 1. Introduction

Heart failure is one of the growing diseases in the United States associated with significant morbidity and mortality. More than 6.5 million people over the age of 20 have heart failure in the United States [1]. According to the Centers for Disease Control and Prevention (CDC), heart failure was listed as a contributing cause of 364,000 deaths in 2018, making it the primary cause of death for approximately 1 in 8 Americans [2]. The mortality rate for heart failure has remained relatively stable over the past decade, with an age-adjusted death rate of 63.2 per 100,000 population in 2019 [2].

Viral infections are a relatively common cause of heart failure exacerbation. Acute respiratory infections caused by viruses, such as influenza or COVID-19, are particularly common causes of heart failure exacerbation. These respiratory infections can cause inflammation and fluid buildup in the lungs, which can increase the workload on the heart and worsen symptoms in patients with pre-existing heart failure.

Since its inception, the Severe Acute Respiratory Syndrome Coronavirus 2 (SARS-CoV-2) pandemic has resulted in substantial morbidity, mortality, and associated healthcare burden across the globe [3]. SARS-CoV-2 has the tendency to affect multi-organ systems due to its systemic hyperinflammatory nature [4]. Prior reported literature have suggested precipitation and worsening of underlying cardiovascular disease in COVID-19 patients [4,5]. The pathophysiology of COVID-19-related cardiac injury is likely multifactorial including direct cytotoxicity, increased myocardial oxygen demand from hyperinflammatory state, and prothrombotic state resulting in intravascular thrombosis [6,7,8,9]. COVID-19 is known to lead to acute heart failure exacerbations (CHF), and frequently, heart failure exacerbation is the presenting symptom of COVID-19 and is associated with worse outcomes [6,10,11,12,13,14,15,16]. In a recent meta-analysis by Vakili et al. involving 6389 infected patients, prevalence of heart failure was 11.50% (95% CI 3.45–22.83) [17].

We conducted our study by utilizing the National Inpatient Sample (NIS) database and compared clinical outcomes in patients with acute congestive heart failure exacerbation with or without concurrent COVID-19. We also assessed outcomes of patients with heart failure with reduced ejection fraction (HFrEF) versus heart failure with preserved ejection (HFpEF) in the setting of COVID-19.

## 2. Materials and Methods

### 2.1. Data Source

We performed a retrospective study utilizing the Agency for Healthcare Research and Quality (AHRQ) 2020 NIS dataset based on hospitalizations from 1 January 2020 to 31 December 2020 [18]. We included all patients 18 years of age and older admitted to the hospital with acute or acute on chronic heart failure exacerbation in the study. We further divided this group based on concurrent diagnosis with COVID-19. We used the International Classification of Diseases 10th—Clinical Modifications (ICD-10-CM) codes to retrieve patient samples with comorbid conditions and ICD-10 procedure codes to identify inpatient procedures. We have provided a detailed code summary in Supplementary Table S1.

### 2.2. Covariates

The NIS data sample includes in-hospital outcomes, procedures, and other discharge-related information. Variables were divided into patient level, hospital level, and illness severity.

- Patient-level: Age, race, sex, comorbidities, income in patient’s zip code, disposition, insurance status.
- Hospital-level: Location, teaching status, bed size, and region.
- Illness severity: Length of stay (LOS), mortality, hospitalization cost, Elixhauser comorbidity score, in-hospital complications, vasopressor use ventilation, and circulatory support.

### 2.3. Study Outcomes

The primary outcome assessed was in-hospital mortality. Secondary outcomes were (a) intubation rate, vasopressor use, sudden cardiac arrest, AKI (acute kidney injury) requiring HD (hemodialysis), and cardiogenic shock, (b) length of stay, (c) financial burden on healthcare, (d) disposition and resource utilization, and (e) in-hospital outcomes of COVID-19 positive patients with acute/acute on chronic heart failure with reduced ejection fraction (HFrEF) vs. COVID-19 positive acute/acute on chronic heart failure with preserved ejection fraction (HFpEF).

### 2.4. Statistical Methods

We utilized STATA 17 (StataCorp LLC, College Station, TX, USA) for statistical analysis. The unweighted sample was 6.47 million observations, and the weighted sample was around 32.3 million discharges for the year 2020. Patients who were admitted with acute or acute on chronic heart failure exacerbation were retrieved with ICD-10 CM codes. We further divided this group based on COVID-19 status. We used the chi-square test to compare categorical variables and linear regression for continuous variables. We used univariate logistic regression to calculate the unadjusted odds ratio for variables of interest for the primary outcome. We used *p* values of ≤0.2 on univariate logistic regression to build a multivariate logistic regression model to adjust for potential confounders. The multivariate linear regression model was used for continuous variables (LOS and total hospital discharge). A two-tailed *p* value of 0.05 was considered to be significant. Subgroup analysis between COVID-19 positive acute/acute on chronic HFrEF vs. COVID-19 positive acute/acute on chronic HFpEF was conducted with the method described above.

## 3. Results

### 3.1. Demographics and Baseline Comorbidities

We identified 2,101,980 patients with acute congestive heart failure (CHF) between 1 January to 31 December 2020. Out of these patients, 75,215 had a diagnosis of COVID-19 (3.6%). COVID-19 patients with acute congestive heart failure were noted to be significantly older (62.1% of patients were above the age of 70 years vs. 57.7%, *p* < 0.001), had a greater proportion of Hispanics (13.7% vs. 7.2%, *p* < 0.001) and African-Americans (20.3% vs. 18.2%, *p* < 0.001), and were more likely to have household incomes below USD 50,000 (35.8% vs. 32.2%, *p* < 0.001) when compared to non-COVID-19 patients (Table 1).

The non-COVID-19 cohort had a higher proportion of patients with cardiac comorbidities including coronary artery disease (50.9% vs. 45%, *p* < 0.001), previous MI (13.8% vs. 11.2%, *p* < 0.001), history of prior PCI (1.3% vs. 1%, *p* = 0.010), and history of CABG (10.7% vs. 10.1%, *p* = 0.012). There was no difference between non-COVID-19 patients and COVID-19 patients regarding uncomplicated diabetes mellitus (10.4% vs. 10.9%, *p* = 0.05) or obesity (28.1% vs. 27.7%, *p* = 0.33). Patients with congestive heart failure and COVID-19 had higher proportions of complicated diabetes mellitus (43.7% vs. 38.3%, *p* < 0.001), chronic kidney disease (29.3% vs. 23.8%, *p* < 0.001), hypertension (1.5% vs. 1.2%, *p* = 0.002), and peripheral artery disease (3.4% vs. 0.5%, *p* < 0.001) (Table 1).

There was no difference between the COVID-19 with CHF vs. non-COVID CHF cohorts in terms of the geographic distribution of the patients, admission to teaching vs. non-teaching, and urban vs. rural hospital status. Moreover, the COVID-19 cohort had a higher proportion of Medicare beneficiaries (74.5% vs. 17.7%, *p* < 0.001) when compared to the non-COVID-19 cohort (Table 1).

### 3.2. In-Hospital Mortality

After multivariate adjustment, patients who presented with acute CHF and COVID-19 infection were associated with significantly higher in-hospital mortality compared to the acute CHF cohort without COVID-19 infection (25.8% vs. 5.5%, adjusted OR 6.3, 95% CI 6.05–6.62, *p* < 0.001) (Table 2). We also examined subgroup mortality and found that among the COVID-19 with acute CHF presentation, Hispanics (15.6% vs. 7.42%, *p* < 0.001) and African Americans (18.1% vs. 13.7%, *p* < 0.001) had an increased in-hospital mortality when compared to the non-COVID-19 cohort. Conversely, a higher proportion of Caucasians in the non-COVID-19 group had a higher inpatient mortality compared to the COVID-19 group (75.7% vs. 62.5%, *p* < 0.001). Patients with age > 70 years hospitalized with acute CHF exacerbation with COVID-19 also had higher mortality when compared to patients in similar age groups without COVID-19 (71% vs. 67.6%, *p* < 0.001) (Table 3).

Moreover, to account for the month-to-month variability in COVID-19 disease burden and the logistical challenges during the early pandemic, we examined monthly mortality among CHF patients with and without COVID-19 (Table 4). The acute CHF and COVID-19 infection were associated with significantly higher in-hospital mortality in the early months of the pandemic in the US (March 2020) compared to the acute CHF cohort without COVID-19 infection adjusted OR 10, 95% CI 8.0–12.5, *p* < 0.001, and lowest in the month of December 2020 adjusted OR 5, 95% CI 4.5–6, *p* < 0.001. The mortality odds downtrended each month as seen in Table 4. Notably, while the earliest reported COVID-19 cases in the US were in late January 2020, we restricted our analysis to March 2020 onwards, since our study relies on ICD-10 codes, which were not reliably available during the early pandemic.

### 3.3. In-Hospital Complications

Patients who presented with COVID-19 with acute CHF exacerbation required more mechanical ventilation (31.3% vs. 17.1%, adjusted OR: 2.3 (95% CI 2.25–2.44, *p* < 0.001)) and had higher vasopressors use (4.9% vs. 2.5%, adjusted OR: 2.06 (95% CI 1.86–2.27, *p* < 0.001)) despite the non-COVID-19 group having higher rates of cardiogenic shock (4.8% vs. 4.2%, adjusted OR: 0.86 (95% CI 0.77–0.93, *p* < 0.001)) and need for mechanical circulatory support (1.5% vs. 0.7%, adjusted OR: 0.44 (95% CI 0.34–0.55, *p* < 0.001)). Patients with COVID-19 and acute CHF exacerbation also had significantly higher rates of acute kidney injury requiring hemodialysis (5.6% vs. 2.9%, adjusted OR: 1.92 (95% CI 1.77–2.09, *p* < 0.001)) and sudden cardiac arrest (5.7% vs. 2.9%, adjusted OR: 1.95 (95% CI 1.79–2.12, *p* < 0.001)).

### 3.4. In-Hospital Quality Measures and Disposition

**Figure 1:**
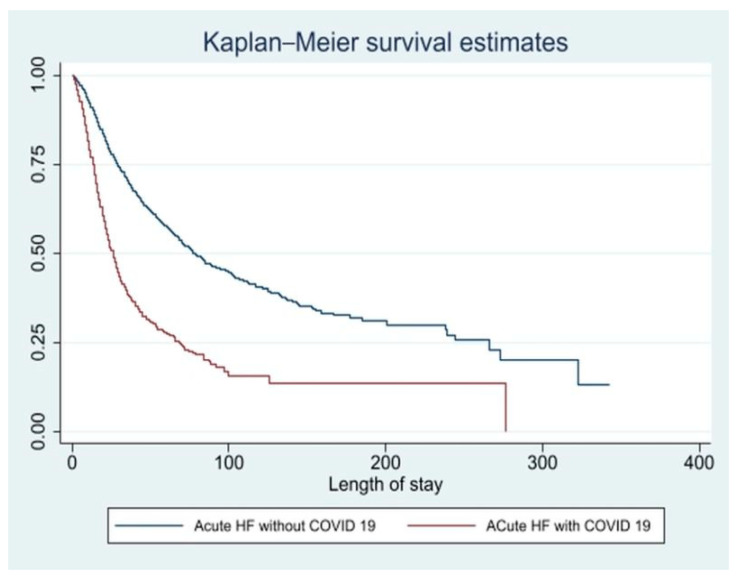
Kaplan–Meier survival analysis of mortality and length of stay.

Patients with COVID-19 and acute CHF exacerbation had increased mean length of stay (11.4 days vs. 6.9 days, adjusted length of stay of 4.5 days higher, *p* < 0.001) compared to acute CHF patients without COVID-19. They had higher mean total hospitalization cost (USD 141,496 vs. USD 96,346, adjusted total cost USD 45,018 higher *p* < 0.001). Of those patients who survived, fewer patients in the COVID-19 with acute CHF cohort were able to return home (36.6% vs. 46.9%, *p* < 0.001) and required skilled nursing or long-term acute care compared to the non-COVID-19 cohort (37.8% vs. 22.1%, *p* < 0.001) (Table 2). The Kaplan–Meier method was used to report length of stay and the survival curve (Figure 1). The Kaplan–Meier survival graph clearly illustrates that the survival probability of AHF patients with COVID-19 is lower than that of those without COVID-19 (Figure 1). The two curves start to diverge shortly after admission, with the COVID-19 group experiencing a sharper decline in survival probability.

### 3.5. Sub-Group Analysis of HFpEF vs. HFrEF Outcomes

We further analyzed outcomes in COVID-19 positive HFrEF vs. HFpEF patients. (Table 5) Patients with HFrEF had higher incidence of in-hospital mortality (26.87% vs. 24.5%, adjusted OR 1.26 (95% CI 1.16–1.36)). The HFrEF group also had higher risk of vasopressor use (5.65% vs. 3.97%, adjusted OR 1.34 (95% CI 1.11–1.62)), sudden cardiac arrest (6.84% vs. 4.43%, adjusted OR 1.46 (95% CI 1.25–1.72)), cardiogenic shock (6.86% vs. 1.04%, adjusted OR 6.59 (95% CI 4.96–8.79)), and need for mechanical circulatory support (1.2% vs. 0.13%%, adjusted OR 8.27 (95% CI 3.66–18.66)). There was no significant difference between the two groups regarding mechanical ventilation needs (32.54% vs. 29.77%, adjusted OR 1.06 (95% CI 0.98–1.14)) and acute kidney injury requiring hemodialysis (5.81% vs. 5.28%, adjusted OR 0.94 (95% CI 0.80–1.1)). The HFrEF group had shorter length of stay when compared to the HFpEF group (11.2 days vs. 11.5, adjusted length of stay 0.8 days lower, *p* < 0.001) but there was no significant difference between mean total hospitalization charge (USD 146,910 vs. USD 135,172, adjusted total charge USD 4411 lower, *p* = 0.282).

## 4. Discussion

In this retrospective analysis, we assessed outcomes of patients with acute congestive heart failure exacerbation with and without concomitant coronavirus disease-19 (COVID-19) infection. We identified 2,101,980 patients hospitalized with the diagnosis of acute congestive heart failure exacerbation (CHF) between 1 January to 31 December 2020. Of these, 75,215 (3.6%) were COVID-19 positive.

To date, most of the prevailing literature has described outcomes of COVID-19 patients with baseline heart failure. To our knowledge, our study is the largest study comparing the outcomes of AHF-COVID+ with AHF-COVID− patients. The prevalence of HF exacerbation among COVID-19 patients stems from studies with limited sample sizes. A meta-analysis by Younas et al. reported pooled prevalence of HF in COVID-19 as 9%, which includes studies comprising patients with known HF and new onset HF with COVID-19 [19]. We report the prevalence of AHF-COVID+ as 3.5% which corresponds to prior reported studies by Rey et al. and Kerolos et al. as 2.5% and 2.9%, respectively [20,21]. Similarly, AHF-COVID+ predominantly affected African-Americans, Medicare beneficiaries, and the elderly population (mean age > 70 years) with baseline comorbidities such as diabetes and kidney disease, which is consistent with prior reported literature [20,22,23]. These disparities can be explained due to lower socio-economic status, inequalities in access to healthcare, and lack of trust in the healthcare system as a whole [24].

Heart failure exacerbation is associated with increased mortality by up to 3.2%, hence a concomitant COVID-19 is likely to worsen the outcomes further [11]. Mehra et al. reported that COVID-19 patients with baseline heart failure had in-hospital mortality of 15.3% vs. 5.6% without heart failure, likely due to poor cardiopulmonary reserve in the setting of hyperinflammatory state seen in HF-COVID+ patients [25]. We report in-hospital mortality of 25.7% in AHF-COVID+ compared to 5.4% in AHF without COVID-19 along with lower survival probability of AHF-COVID+ patients compared to AHF without COVID-19. Bhatt et al. evaluated 8383 AHF-COVID+ patients and reported in-hospital mortality as 24.2%, which is consistent with our study [22]. In contrast, Rey et al. reported in-hospital mortality to be 46.8% in the AHF-COVID+ group [20]. Such a difference is likely explained by our study’s larger sample size and power [20]. The higher mortality in AHF-COVID+ patients is likely attributed to multiple factors such as poor baseline functional status, higher comorbidities, multiorgan dysfunction precipitated by COVID-19 disease (acute respiratory distress syndrome, acute kidney injury, myocarditis, cytokine storm, venous and arterial prothrombotic events), and sociodemographic factors [26]. Moreover, the lack of compliance with guideline-directed medical therapy (GDMT) leads to worse outcomes [20]. Early in the pandemic, it was hypothesized that angiotensin convertase enzyme (ACE) inhibitors might lead to worse outcomes [27,28]. However, further studies demonstrated a low mortality risk with ACE inhibitor and angiotensin receptor blocker use [29,30]. Hence, the American College of Cardiology and the American Heart Association recommended continuing these medications in the absence of evidence of clinical harm. Additionally, mortality associated with AHF-COVID+ could have been mitigated with steroid use and vaccine administration, which were available after June and December 2020, respectively [31,32]. COVID-19 was associated with significant morbidity and mortality at the onset of the pandemic. As noted, the odds of mortality were highest in March 2020, progressively decreasing over the months. The US government took mass-level measures in late March and April 2020, including announcing nationwide emergency mandated masking, social distancing, and restricting unnecessary travel [33]. It was followed by improvement in therapeutics for COVID-19, such as approval of Remdesivir and steroids for emergent use in April and September 2020, respectively. Outpatient treatment and prevention of COVID-19 were aided by the approval of monoclonal antibodies and vaccines in December 2020 [33]. This progressive improvement in the treatment modalities explains the decline in COVID-19 mortality over the months.

Our study showed that 31% of patients with AHF-COVID+ required mechanical ventilation compared to 17% in the AHF-COVID− group. Although, the part difference is likely attributed to COVID-19-associated ARDS as prior studies have reported rates of invasive mechanical ventilation from 29–89% [34]. Garcia et al. reported increased mechanical ventilation up to 22.8% in COVID-19 patients with baseline heart failure compared to 11.9% in COVID-19 without heart failure [30]. Hence, baseline cardiac dysfunction, often accompanied by comorbidities such as kidney dysfunction, increases the risk of respiratory failure necessitating mechanical ventilation.

A multitude of pathological processes can result in a shocking state in COVID-19 such as hypovolemia, vasodilatory state (septic shock, deep sedation, cytokine storm), mechanical ventilation with high positive end-expiratory pressure, pulmonary thromboembolism, and myocarditis [35]. In our cohort, the AHF-COVID+ group was noted to have a higher prevalence of shock requiring vasopressors 4.8% vs. 2.5% in the AHF-COVID− group. However, cardiogenic shock was more prevalent (4.8%) in the HF-COVID− group. Goyal et al. also reported a higher incidence of shock in HF-COVID patients at 15.7% and cardiogenic shock at up to 2.2% [23]. As prior literature reported similar outcomes in regard to shock, hence, we attribute the difference in incidence to the power of our study [23]. Moreover, sudden cardiac arrest is notably higher in our cohort of AHF-COVID+ (5.7%) vs. 2.8% in AHF-COVID−, which is attributed to the higher frequency of shock (primarily vasodilatory and obstructive) [36]. The higher incidence of cardiogenic shock in AHF-COVID− patients can be explained by a higher prevalence of previous myocardial infarction, coronary artery disease, and prior coronary artery bypass graft; hence, they required more mechanical circulatory support up to 2.2% vs. 0.71% in AHF-COVID+ group.

Acute kidney injury (AKI) can be seen with COVID-19 due to cytokine storm-mediated free radical injury, microthrombi in glomerular capillaries, hypovolemia, and cardiac dysfunction [36]. Chatrath et al. reported that COVID-19 patients with HF developed AKI up to 45% vs. 24% in patients without HF [37]. Similarly, Tomasoni et al. and Emad et al. reported the incidence of AKI in COVID-19 patients as 28% and 30%, respectively [38,39]. We report the incidence of AKI as 5.56% in the AHF-COVID+ group vs. 2.9% in the AHF-COVID− group. The difference in reported incidences is again driven by the large sample size and concomitant AHF in our cohort. We hypothesize the higher degree of AKI in HF is attributed to the pathologic processes related to COVID-19.

We report higher hospital length of stay (LOS) in AHF with COVID-19. Alvarez-Garcia et al. reported median hospital LOS in COVID-19 patients with baseline HF as 8 vs. 6 days as compared to COVID-19 patients without HF [30]. Our study includes the whole cohort of COVID-19 hospitalization with varying disease severities which explains the impact of concomitant HF and COVID-19 disease on hospital LOS. Greater disease severity and prolonged hospital LOS increase physical debility and require placement in skilled facilities to improve functionality. This overall creates a substantial financial burden on healthcare systems.

HFrEF is typically associated with an increased risk of heart failure readmissions and mortality compared to HFpEF [40]. Chiocel et al. reported 1-year mortality in HFrEF as 8.8% compared to 6.3% in HFpEF and, notably, a higher prevalence of an underlying ischemic etiology in the former [41]. In our study, subgroup analysis revealed a higher in-hospital mortality in the HFrEF (26.8%) vs. the HFpEF (24.5%) cohort. The higher prevalence of ischemic heart disease in the HFrEF vs. HFpEF group can confer an increased mortality. Our results indicate higher absolute mortality in COVID-19 patients with HFpEF and HFrEF as compared to COVID-19 negative HFrEF and HFpEF patients; the excess mortality is likely attributed to concomitant COVID-19. However, the absolute difference in mortality between COVID-19 patients with HFrEF and HFpEF remains the same, which is consistent with prior reported literature. The incidence of cardiogenic shock was higher in HFrEF patients. Moreover, we report a higher need for mechanical circulatory support, and the incidence of sudden cardiac arrest in the HFrEF subgroup. Similar to our study, Garcia et al. described the rate of cardiogenic shock as 7.8% in COVID-19 patients with HFrEF vs. 2% in HFpEF. Though, no difference in LOS, ICU care, mechanical ventilation, or kidney injury was found between either group, which is also similar with our findings [30]. Bocchi et al. reported that HFrEF patients required higher doses of ionotropic support and mechanical circulatory support in the setting of COVID-19 disease [42]. These findings are explained by poor cardiac contractility to tolerate neurohormonal stress mounted by COVID-19 [26].

Vaccinations against COVID-19 in patients with heart failure are recommended by the major cardiology societies from the USA and Europe [43,44] as they can help reduce morbidity and mortality in this vulnerable population. Jonson et al., in a study consisting of 7094 patients with heart failure, noted a lower likelihood of all-cause hospitalization and mortality in a graded fashion with lower hospitalization and mortality rates in vaccine-boosted individuals and worst in unvaccinated individuals [45]. Future research should include vaccination’s role in influencing outcomes in patients with COVID-19 infection and heart failure.

### Limitations

Data from our retrospective study were collected via the National Inpatient Sample database, which likely has come with inherent selection bias. Acute CHF is a clinical diagnosis and presents similar to acute COVID-19 pneumonia infection, it is a very likely possibility that acute CHF was over/under-diagnosed depending on the provider making the diagnosis. Data from the NIS also does not capture outpatient mortality, so mortality from acute CHF with concurrent COVID-19 infection may be underestimated. Vaccination likely did not affect our results as the first COVID-19 vaccine was made available via EUA on 11 December 2020. It is likely that vaccination for COVID-19 would alter the number of patients with acute CHF and COVID-19, but the prevalence has yet to be studied. Additionally, the NIS does not include lab values and imaging data, so our team had to rely on discharge diagnosis alone. The NIS database also lacks information regarding specific treatment administered to the patients. Acute CHF included in our study is based on ICD-10 codes and is prone to errors; however, larger sample size mitigates the potential coding errors.

## 5. Conclusions

Acute heart failure with COVID-19 is associated with higher in-hospital mortality, lower survival probability, longer hospital stays, and increased healthcare costs compared to patients without COVID-19. Prompt recognition and aggressive management of COVID-19 and heart failure exacerbation is crucial to ensure better outcomes. The approach to treating heart failure in COVID-19 patients remains similar to treatment without COVID-19, however, closer monitoring may be warranted after discharge. Patients with a history of heart failure should be vaccinated and receive optimal heart failure management to prevent hospitalizations. Further prospective studies are needed to identify ways to reduce in-hospital death and improve outcomes in these patients.

## Figures and Tables

**Table 1:** COVID-19 and Acute Congestive Heart Failure (CHF): Unmatched Patient-level characteristics.

Characteristics	Acute CHF without COVID-19	Acute CHF with COVID-19	*p*-Value
*n* = 2,101,980	*n* = 2,026,765 (96.4%)	*n* = 75,215 (3.6%)	
Sex (Female)	46.68%	44.54%	<0.001
Mean age years (SD)			<0.001
Male	69.01 (13.8)	70.3 (14)	
Female	72.96 (13.6)	73.9 (13)	
Age Groups			<0.001
≥18–29	0.61%	0.67%	
30–49	7.06%	6.37%	
50–69	34.67%	30.84%	
≥70	57.66%	62.12%	
Race			<0.001
Caucasians	68.8%	59.59%	
African-American	18.24%	20.34%	
Hispanics	7.77%	13.69%	
Asian or Pacific Islander	2.15%	2.32%	
Native American	0.58%	0.8%	
Others	2.47%	3.27%	
Median Household Income			<0.001
<USD 49,999	32.82%	35.85%	
USD 50,000–64,999	27.88%	27.68%	
USD 65,000–85,999	22.03%	21.35%	
> USD 86,000	17.28%	15.12%	
Insurance Status			<0.001
Medicare	72.7%	74.49%	
Medicaid	11.3%	10.27%	
Private	13.07%	13.43%	
Self-pay	2.93%	1.81%	
Hospital Division			<0.001
New England	4.83%	4.05%	
Middle Atlantic	12.75%	12.9%	
East North Central	16.48%	18.13%	
West North Central	6.41%	6.87%	
South Atlantic	22.26%	20.29%	
East South Central	7.54%	7.05%	
West South Central	11.95%	13.47%	
Mountain	4.86%	5.72%	
Pacific	12.92%	11.52%	
Hospital Bed Size			0.321
Small	22.41%	23.21%	
Medium	28.75%	28.48%	
Large	48.84%	48.31%	
Hospital Teaching Status			0.383
Rural	9.15%	8.81%	
Urban Non-teaching	18.89%	19.45%	
Urban Teaching	71.96%	71.74%	
Elix Sum	6.3 (+/−1.9)	6.3 (+/−1.9)	0.545
Pulmonary Circulation Disorder	22.18%	16.64%	<0.001
Chronic Pulmonary Disease	40.12%	35.45%	<0.001
Diabetes Uncomplicated	10.36%	10.86%	0.053
Diabetes Complicated	38.34%	43.7%	<0.001
Hypothyroidism	18.01%	16.51%	<0.001
Chronic Kidney Disease	23.8%	29.26%	<0.001
Peptic Ulcer Disease (excluding bleeding)	0.65%	0.32%	<0.001
Lymphoma	1.23%	1.06%	0.070
Metastatic Cancer	1.81%	1.02%	<0.001
Solid Tumor Without Metastasis	4.03%	2.45%	<0.001
Rheumatoid Arthritis/Collagen Vascular	3.67%	3.49%	0.267
Obesity	28.07%	27.68%	0.333
Drug Abuse	5.87%	3.73%	<0.001
Hypertension	1.23%	1.51%	0.002
PAD ^a^	0.46%	3.36%	<0.001
OSA ^b^	15.6%	13.1%	<0.001
Liver Disease	6.63%	5.01%	<0.001
Alcohol	4.17%	2.69%	0.095
Smoking	41.64%	9.91%	<0.001
History of PCI ^c^	1.26%	1.02%	0.010
History of CABG ^d^	10.72%	10.05%	0.012
Previous MI ^e^	13.75%	11.16%	<0.001
Coronary Artery disease	50.88%	45.02%	<0.001

Abbreviations: ^a^ PAD: Peripheral Arterial disease; ^b^ OSA: obstructive sleep apnea; ^c^ PCI: Percutaneous Coronary Intervention; ^d^ CABG: Coronary Artery Bypass Graft; ^e^ MI: Myocardial infarction.

**Table 2:** In-hospital outcomes of Acute Congestive Heart Failure (CHF) in COVID-19 Positive and Negative Patients.

Variable	Acute CHF with COVID-19	Acute CHF without COVID-19	*p*-Value
In-hospital Mortality (*n* = 130,310)	25.78% Adjusted odds ratio ^1^	5.47% 6.3 (95% CI 6.05–6.62)	<0.001
Vasopressor Use	4.87% Adjusted odds ratio ^1^	2.54% 2.06 (95% CI 1.86–2.27)	<0.001
Mechanical Ventilation	31.26% Adjusted odds ratio ^1^	17.14% 2.3 (95% CI 2.25–2.44)	<0.001
Sudden Cardiac Arrest	5.73% Adjusted odds ratio ^1^	2.88% 1.95 (95% CI 1.79–2.12)	<0.001
Acute Kidney Injury on HD	5.56% Adjusted odds ratio ^1^	2.94% 1.92 (95% CI 1.77–2.09)	<0.001
Cardiogenic Shock	4.17% Adjusted odds ratio ^1^	4.80% 0.84 (95% CI 0.77–0.93)	0.001
Mechanical Circulatory Support (LVAD ^2^ or pVAD ^3^ or ECMO ^4^)	0.71% Adjusted odds ratio ^1^	1.51% 0.44 (95% CI 0.34–0.55)	<0.001
Mean Total Hospitalization Charge (USD)	USD 141,496 Adjusted total charge ^1^	96,346 USD 45,018 higher	<0.001
Mean Length of Say (days)	11.4 Adjusted length of stay ^1^	6.9 4.5 day higher	<0.001
Disposition			<0.001
Home/Routine	36.62%	46.93%	
SNF ^5^/LTAC ^6^/Nursing Home ^7^	37.79%	22.08%	
Home Health	23.96%	29.07%	
AMA ^7^	1.64%	1.91%	

^1^ Adjusted for age, sex, race, income level, insurance status, discharge quarter, Elixhauser co-morbidities, hospital location, teaching status, and bed size. ^2^ LVAD: Left Ventricular Assist Device; ^3^ pVAD: Percutaneous Ventricular Assist Device; ^4^ ECMO: Extracorporeal Membrane Oxygenation; ^5^ SNF: Skilled Nursing Facility; ^6^ LTAC: Long-Term Acute Care Facility; ^7^ AMA: Against Medical Advice.

**Table 3:** Mortality in Acute Congestive Heart Failure (CHF) in COVID-19 Positive and Negative Patients.

Characteristics	Acute CHF with COVID-19	Acute CHF without COVID	*p*-Value
Gender			0.180
Male	56.5%	55.33%	
Female	43.5%	44.67%	
Race			<0.001
Caucasians	62.51%	75.68%	
African American	18.13%	13.68%	
Hispanics	15.59%	7.42%	
Asian or Pacific Islander	2.63%	2.55%	
Native American	1.2%	0.7%	
Age Groups (years)			<0.001
≥18–29	0.31%	0.48%	
30–49	2.63%	3.9%	
50–69	26.03%	28.01%	
≥70	71.03%	67.61%	

**Table 4:** Monthly Mortality Adjusted Odds Ratio of Acute CHF with COVID-19 compared to Acute CHF patients without COVID-19.

Month	Adjusted Odds Ratio *	*p*-Value
March	10 (8.04–12.53)	<0.001
April	8.48 (7.38–9.74)	<0.001
May	6.41 (5.41–7.60)	<0.001
June	6.74 (5.73–7.93)	<0.001
July	5.64 (4.93–6.46)	<0.001
August	6.18 (5.31–7.20)	<0.001
September	5.61 (4.74–6.65)	<0.001
October	5.86 (5.09–6.74)	<0.001
November	5.32 (4.79–5.90)	<0.001
December	5.0 (4.50–5.56)	<0.001

* Adjusted for age, sex, race, income level, insurance status, Elixhauser co-morbidities, hospital location, teaching status, and bed size.

**Table 5:** In-hospital outcomes in COVID-19 Positive patients with Heart Failure with Reduced Ejection Fraction.

Variable	HFrEF with COVID-19	HFpEF with COVID-19	*p*-Value
COVID-19 Positive with Acute Heart Failure (*n* = 75215)	53.9%	46.1%	
In-hospital Mortality (*n* = 130,310)	26.87% Adjusted odds ratio ^1^	24.50% 1.26 (95% CI 1.16–1.36)	<0.001
Vasopressor Use	5.65% Adjusted odds ratio ^1^	3.97% 1.34 (95% CI 1.11–1.62)	0.002
Mechanical Ventilation	32.54% Adjusted odds ratio ^1^	29.77% 1.06 (95% CI 0.98–1.14)	0.138
Sudden Cardiac Arrest	6.84% Adjusted odds ratio ^1^	4.43% 1.46 (95% CI 1.25–1.72)	<0.001
Acute Kidney Injury on HD	5.81% Adjusted odds ratio ^1^	5.28% 0.94 (95% CI 0.80–1.1)	0.467
Cardiogenic Shock	6.86% Adjusted odds ratio ^1^	1.04% 6.59 (95% CI 4.94–8.79)	<0.001
Mechanical Circulatory Support (LVAD ^2^ or pVAD ^3^ or ECMO ^4^)	1.2% Adjusted odds ratio ^1^	0.13% 8.27 (95% CI 3.66–18.66)	<0.001
Mean Total Hospitalization Charge (USD)	USD 146,910 Adjusted total charge ^1^	USD 135,172 USD 4411 lower	0.282
Mean Length of Stay (days)	11.2 Adjusted length of stay ^1^	11.5 0.8 day lower	<0.001
Disposition			<0.001
Home/Routine	40.9%	31.81%	
SNF ^5^/LTAC ^6^/Nursing Home	34.4%	41.6%	
Home Health	22.51%	25.58%	
AMA ^7^	2.19%	1.01%	

^1^ Adjusted for age, sex, race, income level, insurance status, discharge quarter, Elixhauser co-morbidities, hospital location, teaching status, and bed size. ^2^ LVAD: Left Ventricular Assist Device; ^3^ pVAD: Percutaneous Ventricular Assist Device; ^4^ ECMO: Extracorporeal Membrane Oxygenation; ^5^ SNF: Skilled Nursing Facility; ^6^ LTAC: Long-Term Acute Care Facility; ^7^ AMA: Against Medical Advice.

## Data Availability

Restrictions apply to the availability of these data. Data were obtained from the National Inpatient Sample database, US.

## Supplementary Materials

The following supporting information can be downloaded at: https://www.mdpi.com/article/10.3390/v15030600/s1, Table S1: Diagnosis and ICD-10 Codes.

## References

[1] Benjamin E.J., Blaha M.J., Chiuve S.E., Cushman M., Das S.R., Deo R., de Ferranti S.D., Floyd J., Fornage M., Gillespie C. (2017). Heart Disease and Stroke Statistics-2017 Update: A Report From the American Heart Association. Circulation.

[2] Centers for Disease Control and Prevention Heart Failure|cdc.gov. https://www.cdc.gov/heartdisease/heart_failure.htm.

[3] Miller I.F., Becker A.D., Grenfell B.T., Metcalf C.J.E. (2020). Disease and healthcare burden of COVID-19 in the United States. Nat. Med..

[4] Zaim S., Chong J.H., Sankaranarayanan V., Harky A. (2020). COVID-19 and Multiorgan Response. Curr. Probl. Cardiol..

[5] Long B., Brady W.J., Koyfman A., Gottlieb M. (2020). Cardiovascular complications in COVID-19. Am. J. Emerg. Med..

[6] Tomasoni D., Italia L., Adamo M., Inciardi R.M., Lombardi C.M., Solomon S.D., Metra M. (2020). COVID-19 and heart failure: From infection to inflammation and angiotensin II stimulation. Searching for evidence from a new disease. Eur. J. Heart Fail..

[7] Esakandari H., Nabi-Afjadi M., Fakkari-Afjadi J., Farahmandian N., Miresmaeili S.-M., Bahreini E. (2020). A comprehensive review of COVID-19 characteristics. Biol. Proced. Online.

[8] Huang Y., Yang C., Xu X.-F., Xu W., Liu S.-W. (2020). Structural and functional properties of SARS-CoV-2 spike protein: Potential antivirus drug development for COVID-19. Acta Pharmacol. Sin..

[9] Siripanthong B., Asatryan B., Hanff T.C., Chatha S.R., Khanji M.Y., Ricci F., Muser D., Ferrari V.A., Nazarian S., Santangeli P. (2022). The Pathogenesis and Long-Term Consequences of COVID-19 Cardiac Injury. JACC Basic Trans. Sci..

[10] Clerkin K.J., Fried J.A., Raikhelkar J., Sayer G., Griffin J.M., Masoumi A., Jain S.S., Burkhoff D., Kumaraiah D., Rabbani L. (2020). COVID-19 and Cardiovascular Disease. Circulation.

[11] Dong N., Cai J., Zhou Y., Liu J., Li F. (2020). End-Stage Heart Failure with COVID-19: Strong Evidence of Myocardial Injury by 2019-nCoV. JACC Heart Fail..

[12] Inciardi R.M., Lupi L., Zaccone G., Italia L., Raffo M., Tomasoni D., Cani D.S., Cerini M., Farina D., Gavazzi E. (2020). Cardiac Involvement in a Patient with Coronavirus Disease 2019 (COVID-19). JAMA Cardiol..

[13] Tavazzi G., Pellegrini C., Maurelli M., Belliato M., Sciutti F., Bottazzi A., Sepe P.A., Resasco T., Camporotondo R., Bruno R. (2020). Myocardial localization of coronavirus in COVID-19 cardiogenic shock. Eur. J. Heart Fail..

[14] Zeng J.-H., Liu Y.-X., Yuan J., Wang F.-X., Wu W.-B., Li J.-X., Wang L.-F., Gao H., Wang Y., Dong C.-F. (2020). First case of COVID-19 complicated with fulminant myocarditis: A case report and insights. Infection.

[15] Shi S., Qin M., Shen B., Cai Y., Liu T., Yang F., Gong W., Liu X., Liang J., Zhao Q. (2020). Association of Cardiac Injury With Mortality in Hospitalized Patients with COVID-19 in Wuhan, China. JAMA Cardiol..

[16] Cizgici A.Y., Zencirkiran Agus H., Yildiz M. (2020). COVID-19 myopericarditis: It should be kept in mind in today’s conditions. Am. J. Emerg. Med..

[17] Vakili K., Fathi M., Pezeshgi A., Mohamadkhani A., Hajiesmaeili M., Rezaei-Tavirani M., Sayehmiri F. (2020). Critical complications of COVID-19: A descriptive meta-analysis study. Rev. Cardiovasc. Med..

[18] Agency for Healthcare Research and Quality NIS Database Documentation. https://hcup-us.ahrq.gov/db/nation/nis/nisdbdocumentation.jsp.

[19] Yonas E., Alwi I., Pranata R., Huang I., Lim M.A., Gutierrez E.J., Yamin M., Siswanto B.B., Virani S.S. (2021). Effect of heart failure on the outcome of COVID-19—A meta analysis and systematic review. Am. J. Emerg. Med..

[20] Rey J.R., Caro-Codón J., Rosillo S.O., Iniesta Á.M., Castrejón-Castrejón S., Marco-Clement I., Martín-Polo L., Merino-Argos C., Rodríguez-Sotelo L., García-Veas J.M. (2020). Heart failure in COVID-19 patients: Prevalence, incidence and prognostic implications. Eur. J. Heart Fail..

[21] Kerolos M.M., Ruge M., Gill A., Planek M.I., Volgman A.S., Du-Fay-De-Lavallaz J.M., Gomez J.M.D., Suboc T.M., Williams K.A., Abusin S. (2022). Clinical outcomes of COVID-19 infection in patients with pre-existing cardiovascular disease. Am. Heart J. Plus.

[22] Bhatt A.S., Jering K.S., Vaduganathan M., Claggett B.L., Cunningham J.W., Rosenthal N., Signorovitch J., Thune J.J., Vardeny O., Solomon S.D. (2021). Clinical Outcomes in Patients with Heart Failure Hospitalized with COVID-19. JACC Heart Fail..

[23] Goyal P., Reshetnyak E., Khan S., Musse M., Navi B.B., Kim J., Allen L.A., Banerjee S., Elkind M.S.V., Shah S.J. (2021). Clinical Characteristics and Outcomes of Adults with a History of Heart Failure Hospitalized for COVID-19. Circ. Heart Fail..

[24] Raifman M.A., Raifman J.R. (2020). Disparities in the Population at Risk of Severe Illness From COVID-19 by Race/Ethnicity and Income. Am. J. Prev. Med..

[25] Mehra M.R., Desai S.S., Kuy S., Henry T.D., Patel A.N. (2020). Cardiovascular Disease, Drug Therapy, and Mortality in COVID-19. N. Engl. J. Med..

[26] Bader F., Manla Y., Atallah B., Starling R.C. (2021). Heart failure and COVID-19. Heart Fail. Rev..

[27] Fang L., Karakiulakis G., Roth M. (2020). Are patients with hypertension and diabetes mellitus at increased risk for COVID-19 infection?. Lancet Respir. Med..

[28] Nicin L., Abplanalp W.T., Mellentin H., Kattih B., Tombor L., John D., Schmitto J.D., Heineke J., Emrich F., Arsalan M. (2020). Cell type-specific expression of the putative SARS-CoV-2 receptor ACE2 in human hearts. Eur. Heart J..

[29] Zhang P., Zhu L., Cai J., Lei F., Qin J.-J., Xie J., Liu Y.-M., Zhao Y.-C., Huang X., Lin L. (2020). Association of Inpatient Use of Angiotensin-Converting Enzyme Inhibitors and Angiotensin II Receptor Blockers with Mortality Among Patients with Hypertension Hospitalized with COVID-19. Circ. Res..

[30] Alvarez-Garcia J., Lee S., Gupta A., Cagliostro M., Joshi A.A., Rivas-Lasarte M., Contreras J., Mitter S.S., LaRocca G., Tlachi P. (2020). Prognostic Impact of Prior Heart Failure in Patients Hospitalized with COVID-19. J. Am. Coll. Cardiol..

[31] The RECOVERY Collaborative Group (2021). Dexamethasone in Hospitalized Patients with COVID-19. N. Engl. J. Med..

[32] Polack F.P., Thomas S.J., Kitchin N., Absalon J., Gurtman A., Lockhart S., Perez J.L., Pérez Marc G., Moreira E.D., Zerbini C. (2020). Safety and Efficacy of the BNT162b2 mRNA COVID-19 Vaccine. N. Engl. J. Med..

[33] CDC Museum COVID-19 Timeline|David J. Sencer CDC Museum|CDC. https://www.cdc.gov/museum/timeline/covid19.html.

[34] Wunsch H. (2020). Mechanical Ventilation in COVID-19: Interpreting the Current Epidemiology. Am. J. Respir. Crit. Care Med..

[35] Michard F., Vieillard-Baron A. (2021). Critically ill patients with COVID-19: Are they hemodynamically unstable and do we know why?. Intensive Care Med..

[36] Mir T., Almas T., Kaur J., Faisaluddin M., Song D., Ullah W., Mamtani S., Rauf H., Yadav S., Latchana S. (2021). Coronavirus disease 2019 (COVID-19): Multisystem review of pathophysiology. Ann. Med. Surg..

[37] Chatrath N., Kaza N., Pabari P.A., Fox K., Mayet J., Barton C., Cole G.D., Plymen C.M. (2020). The effect of concomitant COVID-19 infection on outcomes in patients hospitalized with heart failure. ESC Heart Fail..

[38] Tomasoni D., Inciardi R.M., Lombardi C.M., Tedino C., Agostoni P., Ameri P., Barbieri L., Bellasi A., Camporotondo R., Canale C. (2020). Impact of heart failure on the clinical course and outcomes of patients hospitalized for COVID-19. Results of the Cardio-COVID-Italy multicentre study. Eur. J. Heart Fail..

[39] Abdallah E., Al Helal B., Asad R., Hemida M., Nawar E., Kamal M., Reda M., Baharia A., Galal A., Hassan A. (2021). Incidence and Outcomes of Acute Kidney Injury in Critically Ill Patients with Coronavirus Disease 2019. Saudi J. Kidney Dis. Transpl..

[40] Cheng R.K., Cox M., Neely M.L., Heidenreich P.A., Bhatt D.L., Eapen Z.J., Hernandez A.F., Butler J., Yancy C.W., Fonarow G.C. (2014). Outcomes in patients with heart failure with preserved, borderline, and reduced ejection fraction in the Medicare population. Am. Heart J..

[41] Chioncel O., Lainscak M., Seferovic P.M., Anker S.D., Crespo-Leiro M.G., Harjola V.-P., Parissis J., Laroche C., Piepoli M.F., Fonseca C. (2017). Epidemiology and one-year outcomes in patients with chronic heart failure and preserved, mid-range and reduced ejection fraction: An analysis of the ESC Heart Failure Long-Term Registry. Eur. J. Heart Fail..

[42] Bocchi E.A., Lima I.G.C.V., Biselli B., Salemi V.M.C., Ferreira S.M.A., Chizzola P.R., Munhoz R.T., Pessoa R.S., Cardoso F.A.M., Bello M.V.D.O. (2021). Worsening of heart failure by coronavirus disease 2019 is associated with high mortality. ESC Heart Fail..

[43] Driggin E., Maddox T.M., Ferdinand K.C., Kirkpatrick J.N., Ky B., Morris A.A., Mullen J.B., Parikh S.A., Philbin D.M., Vaduganathan M. (2021). ACC Health Policy Statement on Cardiovascular Disease Considerations for COVID-19 Vaccine Prioritization: A Report of the American College of Cardiology Solution Set Oversight Committee. J. Am. Coll. Cardiol..

[44] Rosano G., Jankowska E.A., Ray R., Metra M., Abdelhamid M., Adamopoulos S., Anker S.D., Bayes-Genis A., Belenkov Y., Gal T.B. (2021). COVID-19 vaccination in patients with heart failure: A position paper of the Heart Failure Association of the European Society of Cardiology. Eur. J. Heart Fail..

[45] Johnson K.W., Patel S., Thapi S., Jaladanki S.K., Rao A., Nirenberg S., Lala A. (2022). Association of Reduced Hospitalizations and Mortality Rates Among COVID-19-Vaccinated Patients with Heart Failure. J. Card. Fail..

