# Supplementary material for: COVID-19 and Heart Failure with Preserved and Reduced Ejection Fraction Clinical Outcomes among Hospitalized Patients in the United States
Source: Viruses. 2023 Feb 22;15(3):600. doi: 10.3390/v15030600 (PMC10053519; doi:10.3390/v15030600)
Supplement: Supplementary file 1 [file viruses-15-00600-s001.zip › viruses-2137994-supplementary.docx]

Table S1. Diagnosis and ICD-10 Codes.

| **Diagnosis** | **ICD-10 code** |
| --- | --- |
| Acute Heart Failure exacerbation | I5021, I5023, I5031, I5033, I5041, I5043 |
| Acute Heart Failure with reduced ejection fraction | I5021, I5023, I5041, I5043 |
| Acute heart failure with preserved ejection fraction | I5031, I5033 |
| COVID 19 | U071, U00, U49, U50, U85, J1282 |
| Mechanical ventilation | 5A1945Z,5A1955Z,5A1935Z,5A09357,5A09457,5A09557 |
| Vasopressor | 3E030XZ,3E033XZ,3E040XZ,3E043XZ,3E050XZ,3E053XZ,3E060XZ,3E063XZ |
| Sudden cardiac arrest | I46, I97 |
| Acute Kidney Injury and hemodialysis | N17, N990,5A1D70Z,5A1D90Z,5A1D80Z,5A1D00Z,5A1D60Z |
| Cardiogenic Shock | R570 |
| Mechanical Circulatory Support | 5A02110, 5A02210, 5A0211D, 02HA3RZ, 5A02116, 5A0221D, 5A1522F, 5A1522G,  5A1522H, 5A15A2F, 5A15A2G, 5A15A2H, 5A15223 |
| Chronic Kidney Disease | N181, N182, N1830, N1831, N1832, N184,N185,N189 |
| Pulmonary Circulation Disorder |  |
| Chronic Pulmonary Disease |  |
| Diabetes Uncomplicated |  |
| Diabetes Complicated |  |
| Hypothyroidism |  |
| Peptic Ulcer Disease (excluding bleeding) |  |
| Lymphoma |  |
| Metastatic Cancer | Data Obtained from elixhauser comorbidity index |
| Solid Tumor Without Metastasis |  |
| Rheumatoid Arthritis/Collagen Vascular |  |
| Obesity |  |
| Drug Abuse |  |
| Hypertension |  |
| Peripheral Arterial Disease |  |
| Obstructive Sleep Apenea |  |
| Liver Disease |  |
| Alcohol |  |
| Smoking | F17, F172, F1720, F17200, F17201, F17203, F17208, F17209, F1721, F17210, F17211, F17213,  F17218, F17219, F1722, F17220, F17221, F17223, F17228, F17229, F1729, F17290, F17291, F17293, F17298, F17299, Z87891 |
| History of PCI1 | Z986, Z9861, Z9862 |
| History of CABG2 | Z951 |
| Previous MI3 | I252 |
| Coronary Artery Disease | I2510, I25111, I25118, I25119, I252, I253, I254, I2541, I2542, I255, I256, I257, I2570, I25700,  I25701, I25708, I25709, I2571, I25710, I25711, I25718, I25719, I2572, I25720, I25721, I25728, I25729, I2573, I25730, I25731, I25738, I25739, I2575, I25750, I25751, I25758, I25759, I2576, I25760, I25761, I25768, I25769, I2579, I25790, I25791, I25798, I25799, I258, I2581, I25810,  I25811, I25812, I2582, I2583, I2584, I2589, I259 |
